# Evaluation of Chinese-Herbal-Medicine-Induced Herb-Drug Interactions: Focusing on Organic Anion Transporter 1

**DOI:** 10.1155/2012/967182

**Published:** 2012-09-04

**Authors:** Chang-Ching Lin, Hsien-Yuan Fan, Chien-Wen Kuo, Li-Heng Pao

**Affiliations:** ^1^School of Pharmacy, National Defense Medical Center, Taipei 114, Taiwan; ^2^Division of Pharmacy, Cheng Hsin General Hospital, Taipei 112, Taiwan; ^3^Department of Biotechnology, TransWorld University, Yunlin 640, Taiwan

## Abstract

The consumption of Chinese herbal medicines (CHMs) is increasing exponentially. Many patients utilize CHMs concomitantly with prescription drugs in great frequency. Herb-drug interaction has hence become an important focus of study. Transporter-mediated herb-drug interactions have the potential to seriously influence drug efficacy and toxicity. Since organic anion transporter 1 (OAT1) is crucial in renal active secretion and drug-drug interactions, the possibility of modulation of OAT1-mediated drug transport should be seriously concerned. Sixty-three clinically used CHMs were evaluated in the study. An hOAT1-overexpressing cell line was used for the *in vitro* CHMs screening, and the effective candidates were administered to Wistar rats to access renal hemodynamics. The regulation of OAT1 mRNA expression was also examined for further evidence of CHMs affecting OAT1-mediated transport. Among all the 63 CHMs, formulae Gui Zhi Fu Ling Wan (GZ) and Chia Wei Hsiao Yao San (CW) exhibited significant inhibitions on hOAT1-mediated [^3^H]-PAH uptake *in vitro* and PAH clearance and net secretion *in vivo*. Moreover, GZ showed concentration-dependent manners both *in vitro* and *in vivo*, and the decrease of rOAT1 mRNA expression indicated that GZ not only inhibited function of OAT1 but also suppressed expression of OAT1.

## 1. Introduction

Traditional herbal medicines (THM) have been widely used as complementary alternative medicines (CAM) in the past few decades and the use of THM is continuously growing as one of the most popular CAM all over the world [1–5].

Among THM, Chinese herbal medicine (CHM) is a well defined and established therapeutic system. The therapeutic concepts of CHM are the use of herbal formulations to balance Yin and Yang, Qi and blood [6]. The approaches of CHM formulations are to combine different herbal compounds to increase or promote therapeutic effectiveness, minimize toxicity and side effects, accommodate the promotion of harmony, and optimize the therapeutic effect of each component [7]. CHM formulations are broadly used in global populations. For example, they are considered to be dietary supplements in the USA and natural health products in Canada and Australia. In Asia, they are used as prescriptive drugs in China, Japan, and Taiwan, where CHM is extensively prescribed for treating various kinds of diseases [3, 7–11].

Since the use of CHM is widespread all over the world, the possibility of herb-drug interactions (potential risks while CHM are taken concomitantly with drugs by patients) should be seriously heeded. Modulation of drug transporters by CHM is a proper example to elucidate herb-drug interactions that are mentioned above. Drug transporters are transmembrane proteins that deliver various endogenous and exogenous compounds across cell membranes. As efflux transporter like P-glycoprotein (P-gp) located on intestinal apical membrane is enhanced by CHM, drugs can be highly pumped out by P-gp, which leads to poor bioavailability of drugs [12, 13]. In fact, modulation of transporter-mediated drugs transport is much more complex; it may affect not only absorption of drugs, but also distribution, metabolism, and elimination [14–17]. Once CHMs modulate transporters, they may influence pharmacokinetic of the concomitantly used drugs and further cause adverse effects.

Active secretion that occurs in proximal tubule of kidney is one of the major pathways of drug elimination. In transporters-mediated active secretion, the organic anion transporter (OAT) system is considered to play an important role because it excretes many phase II metabolites in addition to unconjugated anionic compounds against the concentration gradient. Thus the OAT system eliminates a large number of anionic xenobiotics, including a variety of drugs [18–20].

OAT1 is the first cloned member of the OAT family [21–23]. The mRNA expression of OAT1 is predominant in human and rat kidneys whereas it appears weakly in other organs [22, 24]. In kidney, OAT1 is localized at the basolateral membrane of proximal tubular cells [20, 25]. At first, the function of OAT1 was identified as p-aminohippuric acid (PAH)/dicarboxylate exchanger: OAT1 transports outward directly of dicarboxylate (physiologically, *α*-ketoglutarate) moving down its electrochemical gradient in the exchange for the uptake of PAH against its electrochemical gradient [21–23, 26, 27]. 

OAT1 is multispecific in substrates, from endogenous substances to environmental toxins and drugs. Drugs that are transported by OAT1 include nonsteroidal anti-inflammatory drugs (NSAIDs), antiviral drugs, antibiotics, diuretics, antineoplasmics, antiepileptics, and antihypertensive agents [20, 28–30]. Thus, inhibition of OAT1 may probably diminish renal excretion and increase body accumulation of these drugs, which may cause adverse effects. In Japan, it has been reported that severe bone marrow suppression occurred in patients who were coadministered methotrexate (MTX) and NSAIDs. Inhibition of OAT1-mediated MTX renal excretion by NSAIDs is also considered to play a role in the accumulation and adverse effects of MTX [31, 32]. In contrast to the adverse effects, several studies have reported that OAT1-mediated cellular accumulation of antiviral drugs is the reason of nephrotoxicity; hence, inhibition of OAT1 by NSAIDs or probenecid may, in some cases, reduce nephrotoxicity [15, 33–36]. Besides, OAT1 also transports uremic toxins derived from dietary protein such as indoxyl sulfate, which plays an important role in the progression of renal damage. Therefore, OAT1 is important not only in the transport of drugs, but also in the progression of renal diseases.

 Since OAT1 is crucial in renal active secretion and drug-drug interactions, the possibility of modulation of OAT1-mediated drug transport should be seriously concerned. The gradual increase of popularity of CHM arises more caution on drug safety. Once CHM significantly influences OAT1-mediated renal active secretion, especially while CHMs are coadministered with nonherbal drugs, herb-drug interactions may cause severe adverse effects.

 With different ethnicity and culture, Asians are more prone to be encountered with Chinese herbal medicines. This study focused on the herb-drug interaction with the frequently used CHMs in Taiwan, China, and Japan, since CHMs are used in a great amount for disease treatment and supplementary in the daily diet. The purpose of this study is to evaluate the modulation effects of clinical frequently used CHM on OAT1-mediated transport. First, an hOAT1-overexpressing cell line was used to screen CHM, and then the effective candidates were reaffirmed to see whether they exhibited the same pattern *in vivo*. Finally, this study examined how these CHMs affect OAT1-mediated transport; by functional modulation, expressional regulation, or both. 

## 2. Materials and Methods

### 2.1. Cell Culture

 Control and stably hOAT1 expressing Madin-Darby canine kidney type II (MDCK/hOAT1) cells were kindly provided from Dr. Pritchard's laboratory (National Institute of Environmental Health Sciences, USA). Control MDCK II cells were grown in Eagle's Minimum Essential Medium (EMEM, Sigma-Aldrich, USA) supplemented with 1 mM sodium pyruvate and 10% fetal bovine serum (HyClone, USA); MDCK/hOAT1 cells were grown in the same EMEM medium with an extra 200 *μ*g/mL Geneticin (Invitrogen, USA). While in culture, both cells were grown and maintained in a humidified atmosphere consisting of 5% CO_2_ at 37°C. Cells were split 1/10 every 4 to 5 days. 

### 2.2. Chinese Herbal Medicine Formulae-Plant Materials: Sources, Sampling, Identification, Exaction Acquisition, and Physiochemical Analysis

 All CHM formulae were purchased from Sun Ten GMP pharmaceutical company. Each plant of the CHM formulae was collected from different regions in China. Each plant was preserved as the voucher herbarium specimen and deposited at the Brion Research Institute (BRI, Taipei, Taiwan). The authentication of the plants was identified using microscopic and high-performance liquid chromatography (HPLC). All plant parts were classified according to the Pharmacopoeia of the People's Republic of China [37]. 

 Raw herbs were extracted by decoction using hot distilled water and concentrated using instant spray drying and low-temperature vacuuming. Validations of thin layer chromatography and high-performance liquid chromatography were performed as quality control for CHM single herbs and CHM formulae, respectively.

### 2.3. Extraction of Chinese Herbal Medicine Formulae for *In Vitro* Study

 Clinical frequently used CHM formulae and single herbs were selected as candidates for the *in vitro* screening experiments [38]. CHM formulae were all purchased from a GMP pharmaceutical company (Sun Ten Pharmaceutical Co., LTD, Taiwan). All of the CHM formulae are available market products that are frequently prescribed in hospitals in Taiwan. Extraction of CHM formulae was prepared in a way to simulate the physiological condition when a CHM formula was ingested by oral administration in living organisms; CHM formulae are first exposed in extremely acidic conditions in the stomach and then are flowed into neutral conditions in the duodenum. An appropriate amount of CHM formulae was weighted and prepared at 5 mg/mL in 2 g/L NaCl (pH 1.2, adjusted by 1 N HCl) at 37°C. The suspension was first ultrasonicated for 5 min at 37°C and was adjusted to pH 6.8 with 1 N NaOH. Another 5 min ultrasonication at 37°C was conducted, and then the suspension was centrifuged for 10 min. Finally, the supernatant was filtered through an 0.22 *μ*m filter, and the filtrate was further diluted to 500 *μ*g/mL with Hank's buffered saline solution (HBSS; Sigma-Aldrich, USA).

### 2.4. Functional Assay of hOAT1

 Transfection of hOAT1 expressing stable line in MDCK II cells was established in Dr. Pritchard's laboratory. Apical expression of hOAT1 and low uptake background of organic anions permit cells growing on a solid well plate to screen substances that are involved in hOAT1-mediated transport [39, 40]. The procedures of the PAH uptake study were followed by Zalups and Ahmad studies with minor modifications [41, 42]. First, 5 × 10^5^ MDCK/hOAT1 cells were seeded in 24-well plates with a total volume of 2 mL, and medium were changed at the first 24 hr. After culturing for 48 hr, each well was preincubated with 1 mL of 37°C HBSS supplemented 10 mM 4-(2-hydroxyethyl)piperazine-1-ethanesulfonic acid (HEPES, pH 7.4) for three consecutive 5 min periods. The buffer was then aspirated, and 350 *μ*L of the aforementioned HBSS containing 5 *μ*M [^3^H]-PAH (PerkinElmer, USA) were added to each well in the presence or absence of 200 *μ*M probenecid. Uptake period of [^3^H]-PAH was 1 hr in a 37°C incubator. The reaction was quenched by rinsing each well with 1 mL of the “stopping buffer” which was composed of ice cold HBSS containing 10 mM HEPES (pH 7.4). To determine the cellular uptake of [^3^H]-PAH, 1 mL of 1 N NaOH was added into each well to lyze the cells. The plate was shaken with an orbital shaker overnight (at least 12 h). Subsequently, 700 *μ*L of cell lysate was transferred into a counting vial and neutralized with an equal volume of 1 N HCl. The cell lysate was followed by adding 15 mL of Opti-Fluor (PerkinElmer, USA). The radioactivity was measured with a liquid scintillation counter (2100TR Tricarb, Packard, USA). Total protein of each well was determined by the Bradford method according to the instructions of the protein assay kit (BioRad, USA).

### 2.5. Functional Modulation Effects of CHM on hOAT1

A typical OAT1 substrate, [^3^H]-PAH, was administered to MDCK/hOAT1 cells in the presence or absence of CHM extracts. Frequently used 30 CHM formulae and 33 CHM single herbs were selected for the* in vitro* study. The [^3^H]-PAH uptake velocity differences between control ([^3^H]-PAH treated only) and CHM groups were measured to evaluate if CHM extracts would modulate hOAT1-mediated [^3^H]-PAH uptake. First, 5 × 10^5^ MDCK/hOAT1 cells cultured in 24-well plate were conditioned as described above. Second, 350 *μ*L of the aforementioned HBSS containing 50 *μ*g/mL CHM extract and 2 *μ*M [^3^H]-PAH were added to each well for 1 min exposure at 37°C. At the end of the exposure, each well was rinsed with a 1 mL stopping buffer. The subsequent procedures for the determination of intracellular [^3^H]-PAH and total protein were all the same with PAH uptake experiment as described above. 

### 2.6. Cytotoxicity of CHM on MDCK and MDCK/hOAT1 Cells

Control MDCK II and MDCK/hOAT1 cells were seeded in a 96-well plate with a density of 5 × 10^4^ cells per well. Cells were cultured for 48 hr with medium refreshment at the first 24 hr. Before treating the cells with CHM extracts, each well was washed twice with 37°C HBSS. Two hundred *μ*L of CHM extract at different concentrations (5, 50 and 500 *μ*g/mL) were added to each well and incubated for 48 hr. After treating with CHM extracts, each well was washed once with 37°C HBSS. Then 100 *μ*L of 0.5 mg/mL thiazolyl blue tetrazolium bromide (MTT) were added to each well and incubated for 1 h. Finally, cells were lyzed with 100 *μ*L DMSO for 15 min, and the absorbance was measured at 570 nm by a ELISA reader (Witec AG, German). 

### 2.7. Animals

The animal study was approved by the Institutional Animal Care and Use Committee of the National Defense Medical Center (Taipei, Taiwan). Male Wistar rats weighing 350–400 g (8–10 weeks old) were used in the study. All the animals were housed in a standard animal maintenance facility for at least 1 week with free access to food and water before the experiment. Each CHM was prepared with 10 mL milli-Q water by weighing the recommended dose/weight of human × weight of the rat. Oral gavage of the effective CHM formulations in deionized water (from 10.5–70 mg, please refer to Supplementary 1, available online at doi: 10.1155/2012/967182) was conducted for 7 days. The reason for directly gavaging herbal formulations to animals is to present the study under the real physiological condition, in which the herbal medicine was absorbed in the living organisms. Rats in the control group were given deionized water. Rats in the study groups were given 1 mL CHM formulation and the syringe was washed with another 1 mL milli-Q water to ensure all the formulation was administered. Anesthesia was performed on the eighth day by intraperitoneal (ip) injection of pentobarbital sodium (50 mg/kg). Animals were placed on a thermo-regulated heating blanket throughout the study to maintain body temperature. PE-50 catheters were rinsed with normal saline (NS) and 50 IU heparin in NS for femoral vein and femoral artery cannulation, respectively. After cannulating of both the femoral vein and femoral artery, a priming dose of PAH/inulin (30 mg/kg) in NS was injected through the venous catheter; immediately, a continuous intravenous infusion of PAH/inulin (12 mg/mL) was given at a rate of 1 mL/100 g body weight to reach steady-state by an infusion pump (Harvard Apparatus, USA). Blood samples were taken from femoral artery at predose, 30, 45, and 60 min postdose. Plasma was obtained from centrifugation of blood samples at 13,000 rpm for 10 min. After blood sample collection, the animals were sacrificed with potassium chloride and left kidneys being excised and immediately stored in liquid nitrogen. Concentrations of PAH and inulin in plasma samples were then determined by liquid chromatography tandem mass spectrometry (LC-MS/MS). 

### 2.8. Sample Analysis and Estimation of Rat Renal Hemodynamic

The analysis of PAH and inulin in rat plasma by LC-MS/MS was developed and verified in our laboratory [43]. The steady-state model was proposed without collection of urine samples [44]. The clearance (CL_*x*_) can be determined by the following equation: CL_*x*_ = I_*x*_ ×  I_*Y*_/PL, where I_*X*_ was the concentration of the drugs (PAH and inulin) in the infusion solution, I_*Y*_ was the infusion rate, and PL was the concentration of the drugs in plasma [45]. Since both inulin and PAH are eliminated by the kidney without reabsorption, the effect of reabsorption on renal hemodynamic can be neglected [46–48]. Clearance of PAH net secretion (CL_sec  PAH_), the most important parameter of renal hemodynamic that related to OAT1 in this study, can be estimated by the following equation: CL_sec  PAH_ = CL_PAH_ *－*CL_In_.

### 2.9. mRNA Isolation and cDNA Synthesis

 Total RNA was isolated with Trizol Reagent (Invitrogen, USA) from pulverized rat renal tissue according to the manufacturer's instruction. Total RNA extract was suspended in DEPC-treated water. RNA concentration and purity were estimated by measuring the optical density 260/280 nm. The quality and integrity of RNA was analyzed by agarose gel electrophoresis and the samples were stored at −80°C until used. To avoid genomic DNA contamination, each sample was treated with DNase I (Invitrogen, USA) according to the instruction manual. 

 First-strand cDNA was synthesized using a SuperScript First-Strand Synthesis Kit (Invitrogen, USA) according to the manufacturer's instructions. Reverse transcription (RT) was performed at 42°C for 50 min containing 0.2 *μ*g of RNA, 0.5 *μ*g of oligo (dT)12–18, and 50 U of superScript II RNase H^−^ RT. Subsequently, RT was inactivated by incubation at 70°C for 15 min, followed by treatment with Rnase H at 37°C for 20 min. cDNA was stored at −20°C before PCR.

### 2.10. Reverse Transcriptase-Polymerase Chain Reaction (RT-PCR)

 PCR was carried out using a DNA Engine Peltier Thermal Cycle (BioRad, Foster City, CA, USA). PCR amplification was performed in 33 cycles and the reaction conditions used were initial denaturation for 2 min at 94°C, denaturation for 15 s at 94°C, annealing for 30 s at 57°C, and elongation for 1 min at 68°C. For rOAT1 the primers were 5′-TGGCATAATACCGAAGAGCC-3′ (forward) and 3′-TGCTGCTGTTGATTCTGCTT-3′ (reverse), resulting in a 340-bp product. For GAPDH, the primers were 5′-CGGCAACTTCAACGGCACAGTCA-3′ (forward) and 3′-GGTTTCTCCAGGCGGCATGTCA-5′ (reverse), resulting in a 560-bp product. GAPDH was used as a control for variations in the input of RNA. Relative quantity was calculated by the ratio of the gene-specific and the appropriate GAPDH expression. RT-PCR products were resolved by electrophoresis in 1% agarose gel stained with ethidium bromide and visualized under ultraviolet light. The RT-PCR products generated with primers for rOAT1 were tested by sequencing (MB Mission Biotech, Taiwan) and were found to represent the predicted parts of the respective mRNAs.

### 2.11. Statistical Analysis

 The statistical analysis was performed by one-way analysis of variance (ANOVA). Values are expressed as mean ± standard error (SE). *P* values less than 0.05 were considered significant. All analyses were performed using the Statistical Package Social Sciences software (SPSS 12, 2003, SPSS Inc, USA).

## 3. Results

### 3.1. Uptake of PAH

 The uptake velocity of [^3^H]-PAH in MDCK/hOAT1 and control MDCK II cells in the presence or absence of OAT1 inhibitor, probenecid, was evaluated. For MDCK/hOAT1 cells, the rate was 9.94 ± 0.79 pmol/mg protein/min for treating with [^3^H]-PAH only, whereas the addition of probenecid dramatically diminished the rate to 0.54 ± 0.01 pmol/mg protein/min. On the other hand, control MDCK II cells only showed a rate of 0.10 ± 0.01 pmol/mg protein/min, which was almost the same even in the presence of probenecid. The results of both control MDCK II cells and MDCK/hOAT1 cells were similar to Zalups and Ahmad studies [41, 42], proving the function of hOAT1 in the plasma membranes and the repeatability of the experiment in our laboratory.

**Figure 1:**
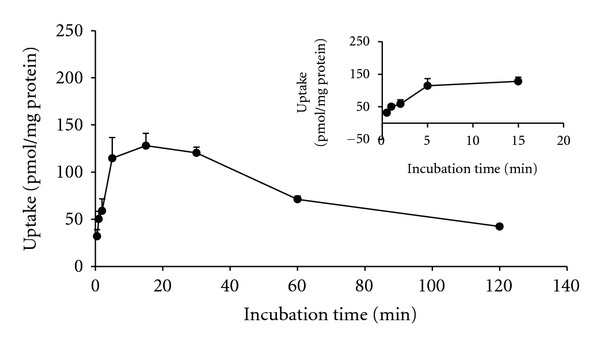
Time-dependent kinetics for the uptake of [^3^H]-PAH in MDCK/hOAT1 cells. The cells were incubated with 2 *μ*M [^3^H]-PAH in the periods indicated at 37°C. The results were expressed as mean ± SE (*n* = 3).

 To determine a suitable uptake period of [^3^H]-PAH in the following CHM screening experiments, a time-dependent kinetic of [^3^H]-PAH uptake in MDCK/hOAT1 was conducted. The result showed that the uptake velocity of [^3^H]-PAH maintained linear before 1 min (Figure 1). Therefore, the uptake velocity of [^3^H]-PAH at 1 min in MDCK/hOAT1 is defined as the initial uptake rate. In the experiments of hOAT1 functional modulation by CHM, [^3^H]-PAH and CHM extract mixture was administered to MDCK/hOAT1 cells and was exposed for 1 min.

An overshoot of [^3^H]-PAH uptake in MDCK/hOAT1 cells is observed. The phenomenon was also reported and explained by Lu et al. [24] and Ueo et al. [49], who used hOAT1 transfected HeLa cells and HEK cells, respectively. The overshoot phenomenon is consistent with exchange-mediated secondary active transport in which an outwardly directed gradient for a cytosolic exchange partner (likely *α*-ketoglutarate) is depleted during the uptake experiment because of an abundance of the external exchange partner.

### 3.2. CHM *In Vitro* Screening

**Figure 2:**
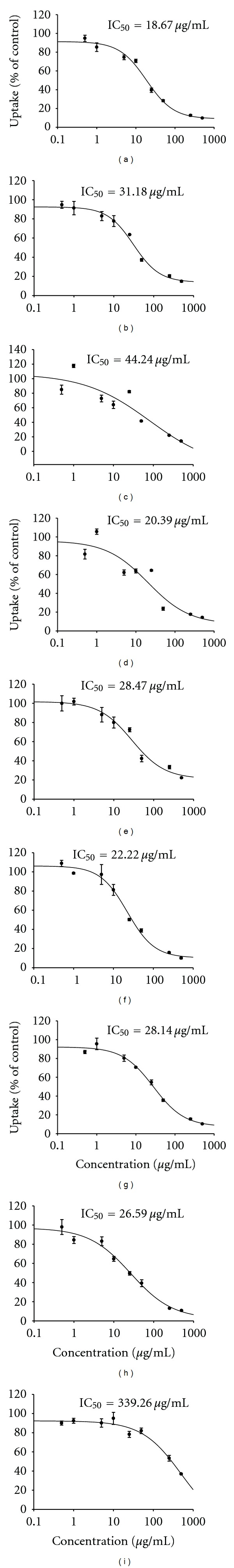
Concentration-dependent effects of (a) GZ, (b) LW, (c) CW, (d) CC, (e) CP, (f) HC, (g) LT, (h) HQ, and (i) HL on the uptake of [^3^H]-PAH in MDCK/hOAT1 cells. The cells were incubated with 2 *μ*M [^3^H]-PAH in the presence of CHM extracts at 0.5, 1, 5, 10, 25, 50, 250, and 500 *μ*g/mL for 1 min at at 37°C. The results were expressed as mean ± SE (*n* = 3).

To evaluate the possibility of OAT1-mediated herb-drug interactions *in vitro*, the extracts of frequently used 30 CHM formulae and 33 single herbs had been tested to estimate whether clinical used CHM formulations would influence hOAT1-mediated [^3^H]-PAH uptake. The results of CHM formulae and CHM single herbs were summarized in Tables 1 and 2, respectively. Interestingly, almost all the tested CHM formulae had significant inhibition on hOAT1 function, while no enhancement of hOAT1 function was observed. Seven of the CHM formulae—Gui Zhi Fu Ling Wan (GZ), Liu Wei Ti Huang Wan (LW), Chia Wei Hsiao Yao San (CW), Chi Chu Ti Huan Wan (CC), Chih Po Ti Huang Wan (CP), Hsin I Ching Fei Tang (HI), and Lung Tan Hsieh Kan Tang (LT)—showed over 50% significant inhibition of hOAT1 uptake ability. In CHM single herbs, half showed significant inhibition on hOAT1 function, but most were about 20–30% inhibition. Only Huang Qin (HQ) had about 50% functional inhibition on hOAT1. Also, two of the CHM single herbs—Jie Geng and Gan Cao—were found to slightly enhance hOAT1-mediated [^3^H]-PAH uptake. Furthermore, seven CHM formulae and two CHM single herbs of the most effective hOAT1 inhibitors showed concentration-dependent manner on hOAT1 functional inhibition (Figure 2). Overall, it seems that CHM single herbs are not so effective on the functional inhibition of hOAT1as CHM formulae are.

### 3.3. Cytotoxicity of CHM Extracts on MDCK/hOAT1 and Control MDCK II Cells

**Figure 3:**
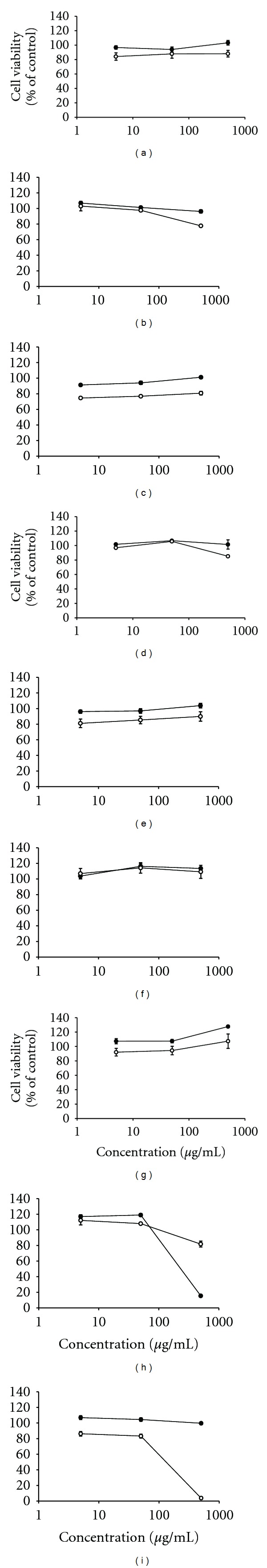
Cytotoxicity of (a) GZ, (b) LW, (c) CW, (d) CC, (e) CP, (f) HC, (g) LT, (h) HQ, and (i) HL on control MDCK II cells (open circle) and MDCK/hOAT1 cells (close circle). Both kinds of cells were seeded in 96-well plate and cultured for 48 hr. CHM extracts at 5, 50, and 500 *μ*g/mL were added into each well and cultured for another 48 hr. Cell viability was evaluated by MTT assay. The results were expressed as mean ± SE (*n* = 3).

**Figure 4:**
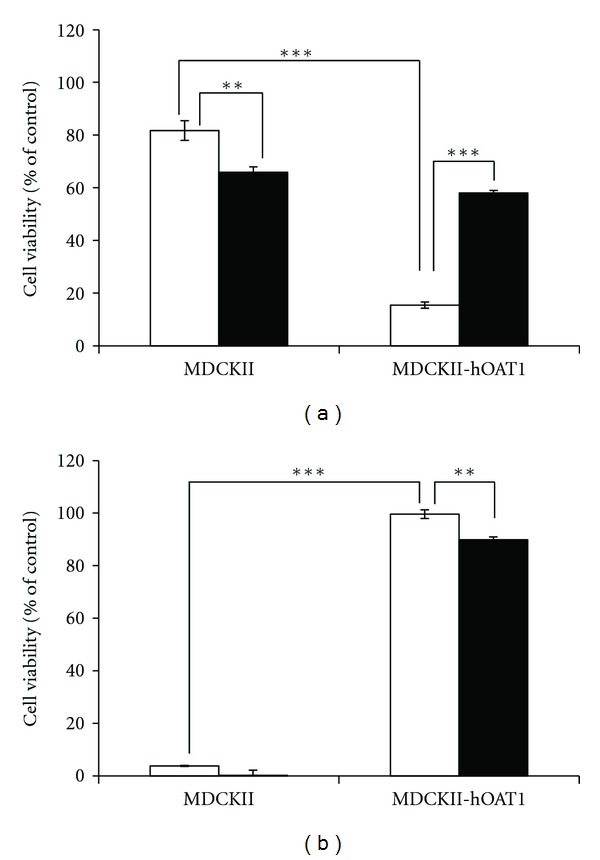
Effects of probenecid on (a) HQ- and (b) HL-induced cytotoxicity in MDCK/hOAT1 cells and control MDCK II cells, respectively. Both kinds of cells were seeded in 96-well plate and cultured for 48 hr. CHM extracts at 500 *μ*g/mL in the presence (dark column) or absence (open column) of 100 *μ*M probenecid were added into each well and cultured for another 48 hr. Cell viability was evaluated by MTT assay. The results were expressed as mean ± SE (*n* = 3).

 To evaluate whether the selected CHM have potential hOAT1-induced nephrotoxicity, various concentrations (5, 50 and 500 *μ*g/mL) of CHM extracts were incubated with MDCK/hOAT1 and control MDCK II cells, and cytotoxicities of CHM extracts on the two types of cells were then evaluated. The result showed that CHM formulae were innoxious to MDCK/hOAT1 cells, while it seemed that CHM formulae were harmful to control MDCK II cells. Comparing to MDCK/hOAT1 cells, LW at 500 *μ*g/mL and CW showed significant reduction of cell viability in control MDCK II cells (Figure 3). Similar results were observed in CHM single herbs at 5 and 50 *μ*g/mL; the cell viabilities of MDCK/hOAT1 cells were higher than control MDCK II cells when administrating the cells with Huang Qin (HQ) and Huang Lien (HL). However, HQ and HL at high concentration (500 *μ*g/mL) presented totally opposite outcomes between MDCK/hOAT1 and control MDCK II cells. In HQ experiment, control MDCK II cells maintained about 80% cell viability when treating cells with high concentration of HQ; on the contrary, MDCK/hOAT1 cells showed dramatica reduction of cell viability in the presence of the same concentration of HQ. A similar phenomenon was observed in an HL high-concentration experiment, whereas it was control MDCK II cells which were killed by HL but not MDCK/hOAT1 cells (Figure 3). Cytotoxicity of HQ in MDCK/hOAT1 cells could be reversed by 100 *μ*M of probenecid; nevertheless, that of HL in control MDCK II cells was irreversible by the same treatment of probenecid (Figure 4).

### 3.4. Effects of CHM on Rat Renal Hemodynamic

To examine whether the *in vitro* hOAT1 inhibitors also influence renal secretion *in vivo*, positive control (cisplatin, ip 5 mg/kg) and effective hOAT1 inhibitors (GZ, LW, CW, CC, CP, HI, LT, HQ, and HL) were administered to Wistar rats for 7 days, and parameters of renal hemodynamic were evaluated. The results were summarized in Table 3. Compared to control group, rats injected with cisplatin exhibited significant reductions in CL_PAH_, CL_In_, and CL_sec  PAH_. On the other hand, CL_PAH_ of GZ and CW were found to have significantly diminished, which could have been caused by the reduction in CL_sec  PAH_. Also, HI and LT showed significant reduction in CL_In_, while CL_PAH_ of both HI and LT were not affected. LW, CP, and HQ were found to enhance CL_sec  PAH_; however, they did not show statistical significance. In the experiment of GZ high dose (HD), dose-dependent manners were observed in CL_PAH_ and CL_sec  PAH_, indicating that elevated dose of CHM should modulate the ability of tubular secretion rather than glomerular filtration.

### 3.5. Regulations of rOAT1 mRNA Expression by CHM

**Figure 5:**
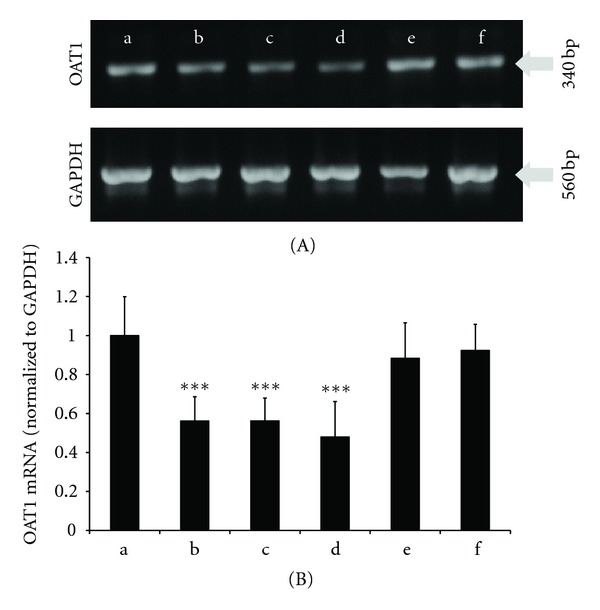
Effects of CHM on mRNA expression of rOAT1 in rat kidney. Total RNA was generated from left kidneys of Wistar rats which were administered CHM formulations or water (control) for 7 days. (A) typical pattern of RT-PCR product; (B) relative mRNA levels of rOAT1; (a) control, (b) cisplatin (positive control, ip), (c) GZ, (d) GZ high dose, (e) LW, and (f) CW. Amount of rOAT1 mRNA signals were normalized to the respective signals from GAPDH. The results were expressed as mean ± SE (*n* = 5-6).

 To obtain direct evidence regarding the role of OAT1 in the renal function inhibited by CHM, the expression of rOAT1 mRNA was evaluated. Kidneys from the control group, cisplatin, GZ, GZ HD, LW (negative control), and CW were excised, and rOAT1 mRNA was evaluated (Figure 5). Compared to control, cisplatin showed significant reduction in rOAT1 mRNA expression. GZ and GZ HD were not only significant downregulation of rOAT1 mRNA expression, but also showed a dose-dependent manner. On the contrary, rOAT1 expression of LW and CW remained steady.

## 4. Discussion

The roles of OAT1 in physiology [50–52], drug induced nephrotoxicity [34, 35, 53, 54], and drug-drug interactions have been studied intensively. However, most of these studies focus on the relationships between OAT1 and synthesized drugs, whereas few focus on that between OAT1 and herbal medicines. The contribution of the presented study is that we are the first ones who evaluated the effects of a large amount of clinical prescribed CHM formulations on OAT1-mediated transport, via *in vitro* screening, *in vivo* hemodynamic monitoring, and OAT1 mRNA expression in rat kidney. The information from our results should be useful in estimating potential herb-drug interactions. 

Phytochemicals were rarely addressed in the presented study because the combinations and interactions of phytochemicals in CHM formulations are too complex to be analyzed. For example, histidine and histamine in Fu Ling (*Poriae cocos*) show activation and inhibition on histamine receptor H1, respectively [7]. One single herb may contain several phytochemicals whereas one formula contains many other single herbs. Synergism and antagonism of phytochemicals within CHM single herbs, and that of CHM single herbs within CHM formulae, make it difficult to evaluate which one plays the major role in herb-drug interactions. Therefore, the strategy of this study is to evaluate CHM that is most commonly prescribed for patients and most ubiquitously used by everyone. The CHM formulations were all purchased from a good manufacturing practice (GMP) pharmaceutical manufacture which ruled out the bias caused by the diversity of the resource, quality, and generating process. Besides, these CHM formulations are extracted and processed by the manufacture to form granulated powder, so that patients can take these CHM formulations directly without further processing procedures. Namely, we are also able to extract these CHM formulations by simulating the condition of the gastrointestinal tract, for these CHM formulations undergo the same pathway in patients.

 Before screening the functional modulation effects of CHM on hOAT1, the uptake velocity of [^3^H]-PAH was evaluated with exactly the same procedures as Zalups and Ahmad [41, 42] previously reported. 

 In the experiment of *in vitro* screening, 25 of 30 CHM formulae and 16 of 33 CHM single herbs were found to significantly inhibit hOAT1-mediated transport. We consider that so many CHM formulations showing their significance may be due to the small standard errors in each group. Therefore, we only selected GZ, LW, CW, CC, CP, HI, LT, HQ, HL (CHM formulae exhibited over 50% inhibition on hOAT1-mediated transport), and HQ, HL (two of the most effective CHM single herbs that inhibited hOAT1-mediated transport) into the following concentration-dependent and cytotoxicity experiments. 

As Figure 2 shows, all the CHM formulations exhibited concentration-dependent manners, and IC_50_ was from 18.67 to 44.24 *μ*g/mL except HL (339.26 *μ*g/mL). On the other hand, an interesting pattern was observed to be a contrast with the recent hypothesis of OAT1 mediated nephrotoxicity; basolateral uptake of substrates from blood into renal proximal tubular cells by OAT1 causes intracellular accumulation of the substrates and further induces tubular damage [33–35, 55]. Should these selected CHM formulations be cytotoxic via OAT1-mediated transport, cell viability of MDCK/hOAT1 cells would be much lower than that of control MDCK II cells. However, we observed a totally opposite phenomenon in the study. That is, cell viability of MDCK/hOAT1 cells was generally higher than control MDCK II cells (except HQ at 500 *μ*g/mL). Also, CHM-formulations-induced cytotoxicity on control MDCK II cells were not concentration-dependent, indicating that there might be a nonspecific factor which injures control MDCK II cells. This implies that it was not associated with OAT1-mediated transport. It is very interesting that at high concentration (500 *μ*g/mL), HQ and HL showed severe toxicity to MDCK/hOAT1 cells and control MDCK II cells, respectively. Cytotoxicity of HQ could be reversed by hOAT1 inhibitor, probenecid, whereas HL could not. Gao et al. [56] found that *Scutellaria baicalensis* (known as HQ) extract was cytotoxic to various lung cancer cells, and cytotoxicity of *Scutellaria baicalensis *might be due to the stoichiometric combination of its three active components, baicalin, baicalein, and wogonin. In this case, HQ extract at 500 *μ*g/mL showed cytotoxic to control MDCK II cells but was much more aggravated in MDCK/hOAT1 cells; therefore, we assume that uptake of active components in HQ extract by hOAT1 may lead to toxic effect as the aforementioned hypothesis. On the other hand, it is hard to figure out why HL extract at 500 *μ*g/mL induced severe cytotoxicity on control MDCK II cells while it was innoxious to MDCK/hOAT1 cells. Although we do not know the possible mechanisms of HL-induced cytotoxicity on control MDCK II cells, it is worth noting that HL at high level in body may cause OAT1-independent nephrotoxicity, since MDCK II cells are renal distal tubular epithelial cells, in which OAT1 did not express. Overall, the *in vitro* cytotoxicity experiment provides information that most CHM formulations tested in the study will not cause cytotoxicity via hOAT1-mediated transport, except HQ at 500 *μ*g/mL. However, the results are incapable of indicating that these CHM formulations are nephrotoxicity-free, for only distal tubular cells were tested in this study. Indeed, it has been suggested that using different cell lines can model differential susceptibilities of corresponding cell types to injury* in vivo *[57, 58]. 

 Intraperitoneal injection of cisplatin, a commonly used model drug that induces rat nephrotoxicity [59–61], is served as positive control to ensure the feasibility of the whole animal experimental system and our new LC-MS/MS analytical method. The result was satisfied that significant reductions of whether CL_PAH_, CL_In_, or CL_sec  PAH_ were observed (Table 3). Cisplatin also inhibited the expression of rOAT1 in kidney (Figure 5), but the downregulation was not so effective as Dan et al. [62] and Morisaki et al. [63] reported. The reason may be that Dan et al. and Morisaki et al. used higher dose of cisplatin (10 mg/kg) than us (5 mg/kg) for injection. On the other hand, GZ was observed to significantly inhibit CL_PAH_, CL_sec  PAH_, and expression of rOAT1 mRNA, indicating that the reduction of renal function by GZ might be due to not only functional inhibition of OAT1 but also downregulation of OAT1 expression in the kidney. It is also possible that CHM preparations only modulate OAT1-mediated transport without regulating the expression of OAT1. In the case of CW, the *in vitro* data showed 57% inhibition on hOAT1-mediated transport (Table 1), the *in vivo* data showed significant reduction in CL_PAH_ and CL_sec  PAH_ (Table 3), whereas the expression of rOAT1 mRNA remained still in comparison to control group (Figure 5). There are also CHM preparations that present different results from *in vitro* and* in vivo*. For instance, LW, CP, HI, and HQ are the cases that were found to effectively inhibit hOAT1* in vitro* whereas slightly, though nonstatistical significant, enhanced CL_sec  PAH_ *in vivo*. We also selected LW to perform the experiment of RT-PCR to test the existence of system error (the expression of rOAT1 mRNA is significantly down- or upregulated while CL_sec  PAH_ remains steady) and found that the expression of rOAT1 mRNA was not significantly regulated as well as renal hemodynamic. 

 Renal active secretion involves transporters-mediated basolateral uptake and apical efflux. Hence, modulation on these transporters by CHM makes it complex to elucidate the mechanisms of herb-drug interactions *in vivo*. Previous studies have reported that renal excretion of PAH is associated with the expression of OAT1 rather than another important basolateral transporter, OAT3, functioning as well as OAT1 [64]. In this OAT1 focusing study, we chose PAH as an *in vivo* model substrate, meaning that we can neglect the effects of CHM on OAT3 in elucidating parameters of renal hemodynamic* in vivo*, yet it is insufficient to rule out the impact of apical efflux transporters. It has been reported that multidrug resistance protein 4 (MRP4) located at apical side is a novel PAH efflux transporter [65]. As a result, studying modulation of MRP4 by CHM should be worthwhile to clarify the mechanism of potential OAT1-mediated herb-drug interactions in kidney. 

## 5. Conclusion

 In conclusion, from *in vitro* to *in vivo*, we proceeded step by step to narrow down CHM formulations that might potentially induce OAT1-related herb-drug interactions. In such interactions, GZ and CW may reduce renal active secretion through inhibition of OAT1-mediated transport, expression of OAT1, or both. The methodologies of the study should be useful in evaluating modulation of drug transporter by CHM; the results of the study should be helpful in abounding information of CHM safety database.

## Supplementary Material

Summary of rat dose calculated from human dose: the suggesting dosage of CHM formulations for human (we assume a 60 kg adult) followed the instruction of the manufacture. The dosage used in rats (we assume a 350 mg rat) was directly measured by multiplying the daily dose of human by the ratio of rat to human body weight.

## Figures and Tables

**Table 1:** Effect of 30 CHM formulae on [^3^H]-PAH uptake in MDCK II/hOAT1.

Inhibition order	Chinese herbal medicine	[^3^H]-PAH uptake (% of control)^a^	Significance^b^
1	Gui Zhi Fu Ling Wan	25.89 ± 0.85	***
2	Liu Wei Ti Huang Wan	32.62 ± 0.74	***
3	Chia Wei Hsiao Yao San	43.00 ± 1.53	***
4	Chi Chu Ti Huang Wan	43.13 ± 2.40	***
5	Chih Po Ti Huang Wan	44.55 ± 3.21	***
6	Hsin I Ching Fei Tang	45.41 ± 1.19	***
7	Lung Tan Hsieh Kan Tang	47.58 ± 3.85	***
8	Kan Lu Yin	51.02 ± 0.64	***
9	Hsiao Chai Hu Tang	56.44 ± 3.27	***
10	Pan Hsia Hsieh Hsin Tang	57.99 ± 3.45	***
11	Tan Kuei Shao Yao Tang	62.22 ± 1.68	***
12	Chuan Chiung Cha Tiao San	63.25 ± 0.80	***
13	Hsieh Fu Chu Yu Tang	65.15 ± 1.62	***
14	Chang Er San	68.95 ± 5.02	***
15	Xin Yi San	69.40 ± 3.00	***
16	Shao Yao Gan Cao Tang	72.27 ± 4.18	***
17	Yin Qiao San	75.24 ± 2.83	***
18	Shu Ching Huo Hsieh Tang	77.39 ± 3.85	***
19	Ten Wang Pu Hsin Tan	77.56 ± 1.06	***
20	Hsiao Ching Lung Tang	79.29 ± 1.84	***
21	Tu Huo Chi Sheng Tang	80.84 ± 3.92	***
22	Ping Wei San	82.47 ± 2.76	***
23	Pu Chung I Chi Tang	83.43 ± 3.12	***
24	Ko Ken Tang	89.72 ± 1.43	*
25	Ma Hsing Kan Shih Tang	90.77 ± 3.83	*
26	Mai Men Dong Tang	93.15 ± 1.80	
27	San Ju Yin	93.75 ± 4.83	
28	Hsiang Sha Liu Chun Tzu Tang	96.96 ± 7.02	
29	Kuei Pi Tang	97.74 ± 5.91	
30	Huo Hsiang Cheng Chi San	102.63 ± 3.42	

^a^Results are expressed as mean ± SE (*n* = 3).

^b^Statistic is performed by one-way ANOVA with posthoc LSD. *P < 0.05, ***P < 0.001.

**Table 2:** Effect of 33 CHM single herbs on [^3^H]-PAH uptake in MDCK II/hOAT1.

Inhibition order	Chinese herbal medicine	[^3^H]-PAH uptake^a ^ (% of control)	Significance^b^
1	Huang Qin	50.76 ± 0.42	***
2	Huang Lien	62.71 ± 2.93	***
3	Chen Pi	73.43 ± 3.08	***
4	Bai Shouh	74.59 ± 1.22	***
5	Hwang Bs	75.46 ± 3.60	***
6	Huang Qi	77.50 ± 1.07	***
7	Ge Gen	77.52 ± 1.87	***
8	Suan Zao Ren	79.21 ± 2.05	***
9	Sha Ren	79.28 ± 5.33	***
10	Du Zhong	79.90 ± 9.46	***
11	Shiang Fu Zhi	82.50 ± 2.24	**
12	Ren San	83.73 ± 5.30	**
13	Sheng Di Huang	84.60 ± 6.44	*
14	Fu Ling	85.77 ± 1.49	*
15	Tan San	86.17 ± 6.47	*
16	Yi Yi Ren	87.33 ± 1.06	*
17	Chuan Kung	90.13 ± 6.57	
18	Jin Yin Hua	90.41 ± 3.10	
19	Ban Shia	90.60 ± 2.01	
20	Tian Lou Gen	91.09 ± 5.99	
21	San Yao	91.64 ± 1.99	
22	Mai Men Tung	91.89 ± 7.06	
23	Zhi Ke	92.93 ± 0.75	
24	Yan Hu Nan	94.23 ± 2.69	
25	Niu Shi	98.55 ± 4.03	
26	Tang Kuei	98.13 ± 3.17	
27	Bai Zhu	100.48 ± 4.89	
28	Bai Zhi	101.99 ± 2.69	
29	Shing Ren	104.68 ± 3.94	
30	Chai Hu	105.87 ± 5.45	
31	Fang Feng	107.05 ± 4.65	
32	Jie Geng	111.14 ± 5.33	*
33	Gan Cao	115.99 ± 1.42	**

^a^Results were expressed as mean ± SE (*n* = 3).

^b^Statistics: one-way ANOVA, LSD post-hoc; significantly different from control.

(*P < 0.05, **P < 0.05, ***P < 0.001).

**Table 3:** Effect of Chinese herbal medicine on renal hemodynamics.

Treatment	CL_PAH_^a,b,d^	CL_In⁡_^a,b,d^	CL_Sec PAH_^a,b,d^
Control	3.32 ± 0.07	0.99 ± 0.03	2.34 ± 0.05
Cisplatin	2.04 ± 0.31***	0.64 ± 0.08***	1.40 ± 0.24***
Gui Zhi Fu Ling Wan	2.86 ± 0.21*	0.97 ± 0.06	1.89 ± 0.16*
Liu Wei Di Huang Wan	3.57 ± 0.14	1.05 ± 0.03	2.52 ± 0.14
Chia Wei Hsiao Yao San	2.88 ± 0.10*	0.92 ± 0.03	1.96 ± 0.09*
Chi Chu Di Huang Wan	3.16 ± 0.02	0.88 ± 0.04*	2.28 ± 0.02
Chih Po Di Huang Wan	3.43 ± 0.06	0.89 ± 0.03	2.54 ± 0.04
Hsin I Ching Fei Tang	3.28 ± 0.10	0.80 ± 0.03***	2.48 ± 0.08
Lung Tan Hsieh Kan Tang	3.14 ± 0.12	0.83 ± 0.04**	2.31 ± 0.13
Gui Zhi Fu Ling Wan HD^c^	2.70 ± 0.11**	0.92 ± 0.03	1.79 ± 0.10**
Huang Qin	3.50 ± 0.18	0.98 ± 0.03	2.52 ± 0.16
Huang Lien	3.27 ± 0.18	1.00 ± 0.04	2.28 ± 0.15

^a^CL_PAH_ = CL_In _+ CL_Sec PAH_.

^b^Results were expressed as mean ± SE (mL/min/100 g BW) (*n* = 5–7).

^c^HD: high dose.

^d^Statistics: one-way ANOVA, LSD post-hoc; significantly different from control (*P < 0.05, **P < 0.005, ***P < 0.001).
