# Supplementary figures and images for: Evaluation of Chinese-Herbal-Medicine-Induced Herb-Drug Interactions: Focusing on Organic Anion Transporter 1
Source: Evid Based Complement Alternat Med. 2012 Sep 4;2012:967182. doi: 10.1155/2012/967182 (PMC3440032; doi:10.1155/2012/967182)

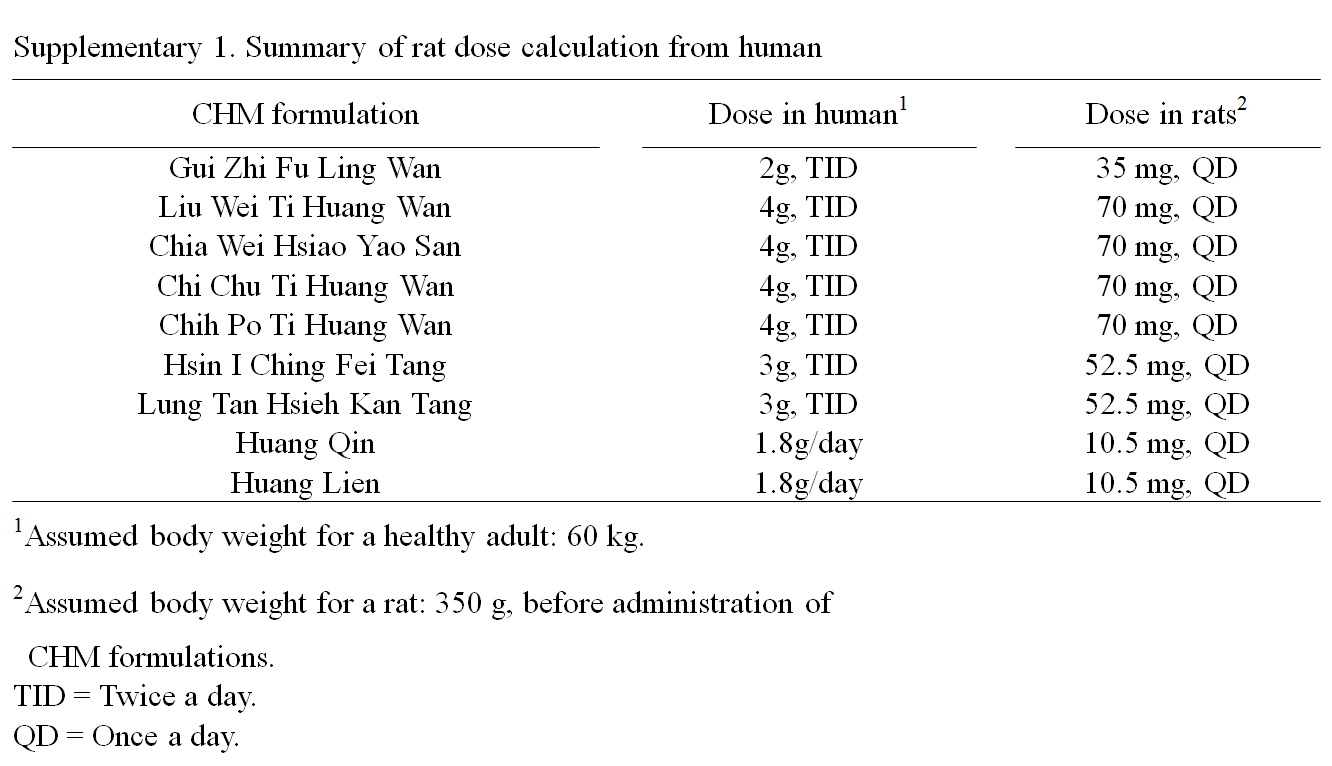

Supplement: Supplementary file 1 — Summary of rat dose calculated from human dose: the suggesting dosage of CHM formulations for human (we assume a 60 kg adult) followed the instruction of the manufacture. The dosage used in rats (we assume a 350 mg rat) was directly measured by multiplying the daily dose of human by the ratio of rat to human body weight. [file 967182.f1.doc]
